# Transcriptome analysis reveals the roles of nitrogen metabolism and sedoheptulose bisphosphatase pathway in methanol‐dependent growth of *Corynebacterium glutamicum*

**DOI:** 10.1111/1751-7915.13863

**Published:** 2021-06-16

**Authors:** Liwen Fan, Yu Wang, Jin Qian, Ning Gao, Zhihui Zhang, Xiaomeng Ni, Letian Sun, Qianqian Yuan, Ping Zheng, Jibin Sun

**Affiliations:** ^1^ School of Life Sciences University of Science and Technology of China Hefei 230026 China; ^2^ Key Laboratory of Systems Microbial Biotechnology Tianjin Institute of Industrial Biotechnology Chinese Academy of Sciences Tianjin 300308 China; ^3^ University of Chinese Academy of Sciences Beijing 100049 China; ^4^ College of Biotechnology Tianjin University of Science and Technology Tianjin 300457 China

## Abstract

Methanol is a promising feedstock for biomanufacturing of fuels and chemicals. Although efforts have been made to engineer platform microorganisms for methanol bioconversion, the substrate uptake and cell growth rates on methanol are still unsatisfactory, suggesting certain limiting factors remain unsolved. Herein, we analysed the global metabolic regulation changes between an evolved methanol‐dependent *Corynebacterium glutamicum* mutant and its ancestral strain by transcriptome analysis. Many genes involved in central metabolism including glycolysis, amino acid biosynthesis and energy generation were regulated, implying the adaptive laboratory evolution reprogrammed the cellular metabolism for methanol utilization. We then demonstrated that nitrate could serve as a complementary electron acceptor for aerobic methanol metabolism, and the biosynthesis of several amino acids limited methylotrophic growth. Finally, the sedoheptulose bisphosphatase pathway for generating methanol assimilation acceptor was found effective in *C. glutamicum*. This study identifies limiting factors of methanol metabolism and provides engineering targets for developing superior synthetic methylotrophs.

## Graphical abstract

Transcriptome analysis of methanol‐dependent *Corynebacterium glutamicum* reveals metabolic regulation changes in central metabolism. It is experimentally verified that nitrate could serve as an additional electron acceptor for aerobic methanol metabolism and the biosynthesis of several amino acids limits methylotrophic growth, and the SBPase variant for Ru5P regeneration is effective in methylotrophic *C. glutamicum*.

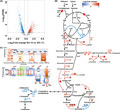

## Introduction

With the development of biotechnology and the pursuit of green and sustainable development, methanol has recently gained increasing attention on replacing sugars and agricultural products to participate in biomanufacturing (Wang *et al*., 2020b). Methanol, the simplest saturated unitary alcohol, can be produced from various carbon‐containing feedstocks, such as syngas, natural gas, carbon dioxide, coals and biomass (Brautaset *et al*., 2007; Rauchle *et al*., 2016). Furthermore, methanol is a kind of highly reduced carbon source, and the degree of reduction per carbon of methanol (κ = 6) is 50% higher than that of glucose (κ = 4) (Zhang *et al*., 2018a,b). Hence, the production of various bulk chemicals including amino acids, organic acids and alcohols using methanol as a substrate has a theoretically higher conversion yield compared with the production processes based on sugar feedstocks (Motoyama *et al*., 1993; Dai *et al*., 2017; Zhang *et al*., 2018a, 2019; Wang *et al*., 2019).

Based on the development of advanced genetic tools for platform microorganisms and the understanding of native methylotrophs, engineering synthetic methylotrophs by transferring the methanol utilization pathway into non‐methylotrophic platform microorganisms is becoming attractive and practical (Haynes and Gonzalez, 2014; Whitaker *et al*., 2015; Zhang *et al*., 2017). The well‐characterized methanol utilization pathway, ribulose monophosphate (RuMP) cycle, overlaps with the typical sugar metabolic pathway, except for three enzymes, methanol dehydrogenase (Mdh), 3‐hexulose‐phosphate synthase (Hps) and 6‐phospho‐3‐hexuloisomerase (Phi). Many efforts have been made on rebuilding the RuMP cycle in platform microorganisms such as *Escherichia coli*, *Corynebacterium glutamicum* and *Saccharomyces cerevisiae*. After the initial success in engineering synthetic methylotrophs to assimilate methanol for biomass and metabolites biosynthesis in the presence of additional carbon sources or nutrients, fully methylotrophic *E. coli* strains have recently been created by combining rational engineering and adaptive laboratory evolution (ALE) (Chen *et al*., 2020; Kim *et al*., 2020). Although these successes provide a promising starting point for methanol‐based biomanufacturing, the methanol uptake rate and cell growth rate on methanol are still not comparable to those of glucose, suggesting that more unknown limiting factors need to be identified and overcome.

Adaptive laboratory evolution combined with omics analysis is a useful strategy to identify non‐intuitive but important targets for strain improvement (Stella *et al*., 2019). In order to establish the direct association between methanol assimilation and cell growth of synthetic methylotrophs, several groups designed methanol‐dependent strains that co‐utilize methanol with an auxiliary carbon source (e.g. xylose, ribose, gluconate and glucose) (Chen *et al*., 2018; He *et al*., 2018; Meyer *et al*., 2018; Tuyishime *et al*., 2018; Bennett *et al*., 2020b; Hennig *et al*., 2020). Since the growth rate of methanol‐dependent strains was positively associated with methanol utilization efficiency, ALE was successfully applied to obtained mutants with improved methanol utilization capability. Subsequent genome analysis combined with reverse metabolic engineering was used to identify key genetic mutations affecting methanol utilization and tolerance. We previously developed a methanol‐dependent synthetic methylotroph based on the important industrial workhorse *C. glutamicum*, which has been used to produce approximately 70 natural and non‐natural compounds including 6 million tons of amino acids per year (Becker and Wittmann, 2012; Becker *et al*., 2018). The engineered strain MX‐10 expressed heterologous xylose isomerase (XylA) from *E. coli*, Mdh from *Bacillus stearothermophilus* DSM 2334, and Hps and Phi from *B. methanolicus*. The native ribose 5‐phosphate isomerase (RpiB) and formaldehyde oxidation enzymes mycothiol‐dependent formaldehyde dehydrogenase (AdhE) and acetaldehyde dehydrogenase (Ald) were deactivated to achieve methanol‐dependent growth with xylose as a co‐substrate (Tuyishime *et al*., 2018). To accelerate cell growth and methanol utilization, the strain MX‐10 was subjected to ALE for 206 days and total of 14 passages in minimal medium were supplemented with methanol and xylose. The evolved mutant MX‐11 exhibited improved methanol‐dependent growth and methanol uptake. Whole‐genome sequencing of the mutant MX‐11 was performed, and mutations involved in regulating uptake of methanol and xylose, redox and energy balance, and methanol tolerance were identified (Tuyishime *et al*., 2018).

In this study, transcriptome profiling of the ancestral methanol‐dependent *C. glutamicum* strain MX‐10 and evolved mutant MX‐11 was conducted to analyse the metabolic regulations that benefit methanol‐dependent growth. The differentially expressed genes involved in glycolysis, pentose phosphate pathway (PPP), amino acid biosynthesis, respiratory chain, etc., in the evolved mutant MX‐11 are suggested to be responsible for the improved methylotrophy. Based on the transcriptome analysis, we further investigated the roles of nitrogen (exogenous nitrate and amino acids) metabolism and sedoheptulose bisphosphatase (SBPase) pathway on methylotrophic growth. This study underscores the importance of redox balance, biosynthesis of limiting amino acids and regeneration of ribulose 5‐phosphate (Ru5P) in methanol metabolism in *C*. *glutamicum* and provides targets for engineering superior synthetic methylotrophs.

## Results and discussion

### Growth test of the ancestral strain MX‐10 and evolved mutant MX‐11

**Fig. 1:**
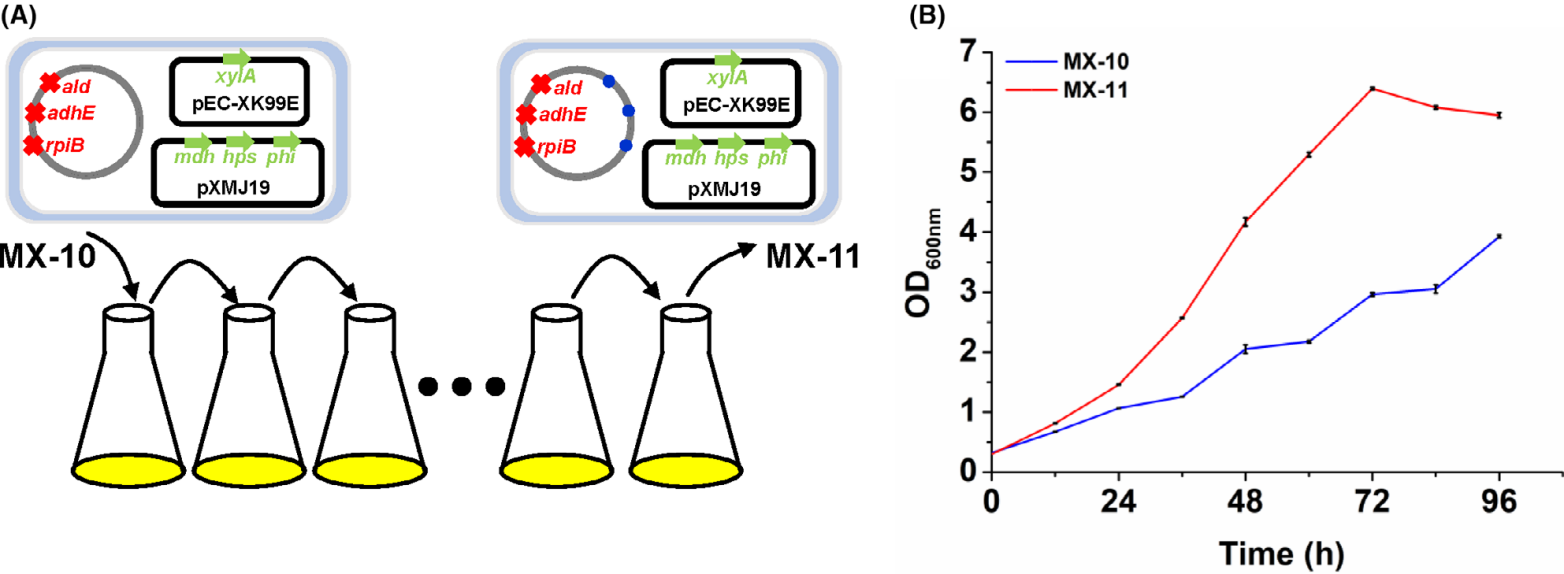
Growth test of evolved methanol‐dependent *C. glutamicum* strain MX‐11 and its ancestral strain MX‐10. A. Schematic illustration of the acquisition of strain MX‐11 by ALE using strain MX‐10 as a starting point. Red crosses represent knock‐out of *rpiB*, *adhE* and *ald* genes. Blue dots represent mutations accumulated during ALE. B. Growth curve of methanol‐dependent *C. glutamicum* strains MX‐10 (blue) and MX‐11 (red) in LB medium supplemented with 4 g l^−1^ methanol and 4 g l^−1^ xylose. Error bars indicate standard deviations from three parallel experiments (*N* = 3).

The methanol‐dependent *C. glutamicum* strain MX‐10 was constructed by disrupting RpiB, AdhE and Ald and overexpressing heterologous RuMP enzymes Mdh, Hps and Phi. Strain MX‐10 was subjected to ALE in CGXII minimal medium with methanol and xylose as the co‐substrates, and an evolved mutant MX‐11 with improved cell growth rate was obtained (Tuyishime *et al*., 2018) (Fig. 1A). Because strain MX‐10 has an exceedingly long and unfixed lag phase in minimal medium (up to 15 days), it is difficult to directly compare the growth characteristics of the ancestral strain MX‐10 and the evolved mutant MX‐11. Such a long lag phase was usually observed for unevolved synthetic methylotrophs in methanol minimal medium (Meyer *et al*., 2018; Chen *et al*., 2020). Chen and colleagues reported the lag phase of methylotrophic *E. coli* could reach as long as 20 days, which was possibly caused by formaldehyde cytotoxicity (Chen *et al*., 2020).

It was reported that supplement of nutrients such as amino acids and vitamins helped to initiate methanol‐dependent growth (Gonzalez *et al*., 2018; Chen *et al*., 2020). Therefore, strains MX‐10 and MX‐11 were cultivated in LB medium containing methanol and xylose. Both strains showed better growth performances in LB‐based medium than CGXII‐based medium, while strain MX‐11 still outperformed MX‐10 (Fig. 1B). Specifically, the specific growth rate of strain MX‐11 at exponential phase was 0.044 ± 0.001 h^−1^, which was approximately 47% higher than its growth rate in CGXII minimal medium supplemented with the same carbon sources (0.030 ± 0.001 h^−1^). In LB‐based medium, strain MX‐11 grew to the highest OD_600 nm_ of 6.40 in 72 h (Fig. 1B), while it grew to a final OD_600 nm_ of 3.82 in 120 h in CGXII‐based medium (Tuyishime *et al*., 2018). Different from the moderate growth improvement for strain MX‐11 in LB‐based medium, growth of strain MX‐10 was dramatically enhanced since almost no lag phase was observed in LB‐based medium. The specific growth rate of strain MX‐10 at exponential phase was 0.027 ± 0.001 h^−1^. The results suggest that the nutrients in LB medium, such as amino acids, vitamins and nucleotides, could efficiently initiate the methanol‐dependent growth of methylotrophic *C. glutamicum* strains, especially the unevolved strain MX‐10. The relatively smaller growth difference in strain MX‐11 in LB‐based and CGXII‐based media suggests the metabolism of strain MX‐11 was well evolved during ALE for methanol metabolism.

### Transcriptome profiling of strains MX‐10 and MX‐11

**Fig. 2:**
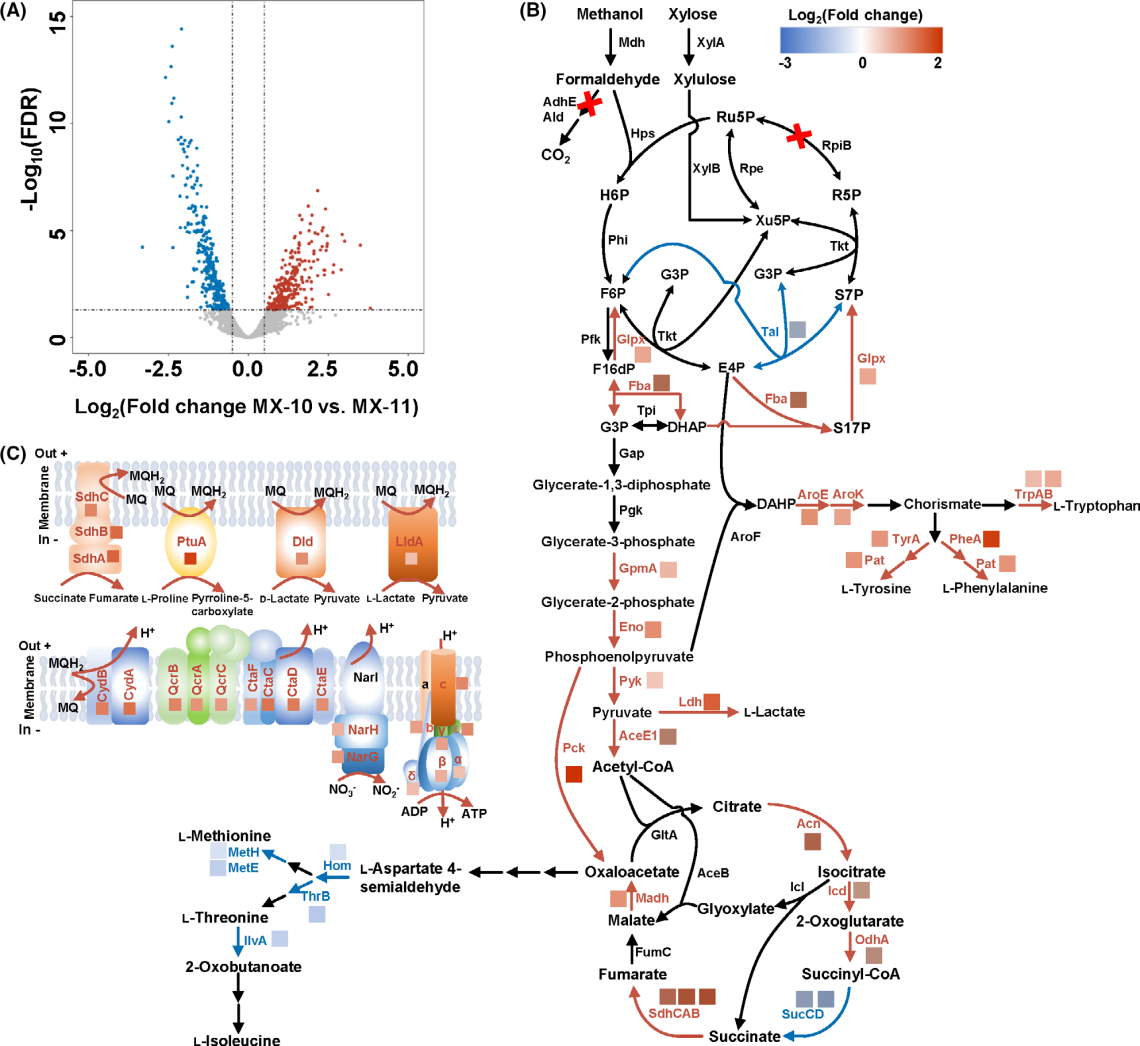
Transcriptome analysis of *C. glutamicum* strains MX‐10 and MX‐11. A. Volcano plot of differential transcription levels as determined by RNA sequencing (*N* = 3). B. mRNA level changes in genes involved in the central metabolism pathways between MX‐11 vs. MX‐10. C. mRNA level changes in genes involved in the respiratory chain between MX‐11 vs. MX‐10. Only significant changes (log_2_(fold change) > 0.5 or < −0.5, FDR<0.05) were shown. Upregulated and downregulated enzymes are indicated with red and blue, respectively. Enzymes: NAD‐dependent methanol dehydrogenase (Mdh), 3‐hexulose‐6‐phosphate synthase (Hps), 6‐phospho‐3‐hexuloisomerase (Phi), mycothiol‐dependent formaldehyde dehydrogenase (AdhE), acetaldehyde dehydrogenase (Ald), xylose isomerase (XylA), xylulokinase (XylB), ribose phosphate isomerase (RpiB), ribulose phosphate epimerase (Rpe), transketolase (Tkt), transaldolase (Tal), fructose 1‐phosphate kinase and related fructose 6‐phosphate kinase (PfkB), fructose bisphosphatase/sedoheptulose bisphosphatase (GlpX), fructose bisphosphate aldolase (Fba), triosephosphate isomerase (Tpi), glyceraldehyde 3‐phosphate dehydrogenase (Gap), phosphoglycerate kinase (Pgk), phosphoglycerate mutase (GpmA), enolase (Eno), pyruvate kinase (Pyk), pyruvate dehydrogenase subunit E1 (AceE1), FMN‐dependent l‐lactate dehydrogenase (LldA), NAD‐dependent l‐lactate dehydrogenase (Ldh), FAD/FMN‐containing d‐lactate dehydrogenase (Dld), phosphoenolpyruvate carboxykinase (Pck), citrate synthase (GltA), aconitate hydratase (Acn), isocitrate dehydrogenase (Icd), α‐oxoglutarate dehydrogenases E1 component (OdhA), succinyl‐CoA synthetase (SucCD), succinate dehydrogenase (SdhCAB), fumarate hydratase (FumC), malate dehydrogenase (Madh), 3‐deoxy‐7‐phosphoheptulonate synthase (AroF), shikimate kinase (AroK), shikimate 5‐dehydrogenase (AroE), tryptophane synthase (TrpAB), prephenate dehydratase (PheA), histidinol‐phosphate aminotransferase (Pat), prephenate dehydrogenase (TyrA), homoserine dehydrogenase (Hom), homoserine kinase (ThrB), methionine synthase Ⅰ (MetH), methionine synthase Ⅱ (MetE), threonine dehydratase (IlvA), nitrate reductase α subunit (NarG), nitrate reductase β subunit (NarH), nitrate reductase γ subunit (NarI), proline dehydrogenase (PtuA), cytochrome *bc*_1_ *c* complex (QcrCAB), cytochrome *aa*_3_ complex (CtaCDEF), cytochrome *bd* oxidase (CydBA), ATP synthase (α, Cgl1210; β, Cgl1212; γ, Cgl1211, δ, Cgl1209; ε, Cgl1213; a, Cgl1206; b, Cgl1208; c, Cgl1207). Metabolites: ribose 5‐phosphate (R5P), ribulose 5‐phosphate (Ru5P), xylulose 5‐phosphate (Xu5P), glyceraldehyde 3‐phosphate (G3P), erythrose 4‐phosphate (E4P), sedoheptulose 7‐phosphate (S7P), fructose 6‐phosphate (F6P), hexulose 6‐phosphate (H6P), fructose 1,6‐bisphosphate (F16dP), dihydroxyacetone phosphate (DHAP), 3‐deoxy‐arabino‐heptulonate 7‐phosphate (DAHP), menaquinone (MQ), menaquinol (MQH_2_).

Although the genome of the evolved mutant MX‐11 has been sequenced and some mutations involved in metabolism of methanol and xylose, balance of redox and energy, and tolerance of methanol were detected, the information is still limited to fully explain the contribution of ALE to improved methanol‐dependent growth (Tuyishime *et al*., 2018; Wang *et al*., 2020a). Transcriptome profiling of strains MX‐10 and MX‐11 was then conducted to decipher the regulation differences. Cells cultivated with CGXII minimal medium containing methanol and xylose were collected at mid‐exponential phase and used for RNA extraction and sequencing. Pearson’s correlation coefficient test and PCA were used to evaluate the accuracy and repeatability of transcriptome analysis. The results show the high accuracy and reliable repeatability of the experimental methods (Fig. S1). A total of 943 genes showed statistically significant expression differences. Among them, 476 and 467 genes were upregulated and downregulated in strain MX‐11, respectively (Fig. 2A and Table S1). These differentially expressed genes were classified into 27 cellular processes on the basis of KEGG_small_class annotation, including amino acid metabolism, carbohydrate metabolism, transcription, translation and energy metabolism. In strain MX‐11, a large proportion of genes falling in the functional categories of carbohydrate metabolism, global and overview maps and energy metabolism were upregulated (Fig. S1).

Specifically, the central metabolic pathways including glycolysis and part of tricarboxylic acid (TCA) cycle were generally enhanced, except for the succinyl‐CoA synthetase (Fig. 2B). Upregulation of glycolysis and part of TCA cycle was expected to fulfil the bioenergetic and biosynthetic requirements of methylotrophic *C. glutamicum*. PPP was essential for the replenishment of formaldehyde assimilation acceptor Ru5P, whereas transcription of the related genes was not significantly changed, except for the transaldolase (Tal) encoding gene (*cgl1575*) that was downregulated in strain MX‐11. Tal catalyses the formation of sedoheptulose 7‐phosphate (S7P) and glyceraldehyde 3‐phosphate (G3P) from fructose 6‐phosphate (F6P) and erythrose 4‐phosphate (E4P), which channels carbon flux for Ru5P formation. Interestingly, fructose bisphosphate aldolase (Fba) encoding gene (*cgl2770*) and bifunction fructose bisphosphatase/sedoheptulose bisphosphatase (GlpX) encoding gene (*cgl1019*) were significantly upregulated. It was reported that Fba and GlpX from *E. coli* can catalyse the conversion of dihydroxyacetone phosphate (DHAP) and E4P to sedoheptulose 1,7‐bisphosphate (S17P) and the dephosphorylation of S17P to S7P, respectively. Overexpression of Fba and GlpX in methylotrophic *E. coli* improved methanol assimilation by activating the SBPase variant for Ru5P regeneration (Woolston *et al*., 2018). However, the SBPase variant activity has not been experimentally verified for GlpX and Fba from *C. glutamicum*.

Different transcription regulations were observed for the biosynthesis pathways of two groups of amino acids (aromatic amino acids and l‐aspartate family amino acids) (Fig. 2B). For biosynthesis of aromatic amino acids, E4P and phosphoenolpyruvate are extracted from PPP and glycolysis, respectively, for biosynthesis of the precursor chorismite, which is used for biosynthesis of l‐tyrosine, l‐phenylalanine and l‐tryptophan. Related genes *aroE* (*cgl1629*), *aroK* (*cgl1622*), *tyrA* (*cgl0226*), *pheA* (*cgl2899*), *trpA* (*cgl3035*), *trpB* (*cgl3034*) and *pat* (*cgl0218*) were upregulated by 2.15‐, 1.88‐, 2.06‐, 3.73‐, 1.61‐, 1.79‐ and 2.08‐fold, respectively. Conversely, genes involved in biosynthesis of several l‐aspartate family amino acids, such as l‐methionine, l‐threonine and l‐isoleucine, were downregulated, which seems contrary to the improved cell growth. It has been suggested that l‐methionine metabolism affects cellular tolerance to methanol. Mutations in l‐methionine metabolic enzymes such as MetY (*O*‐acetyl‐l‐homoserine sulfhydrolase) and MetK (*S*‐adenosylmethionine synthetase) were reported to combat methanol toxicity (Hennig *et al*., 2020; Wang *et al*., 2020a). In strain MX‐11, mRNA levels of *hom*, *metH*, *metE* and *metK* genes involved in methionine metabolism were downregulated. As for *metY* gene, its mRNA level was not significantly changed compared with that of strain MX‐10. Considering the different transcription regulations, it will be interesting to investigate the effect of amino acid metabolism on methanol‐dependent growth. Energy supply plays an important part in cellular metabolism processes, such as protein synthesis and degradation, stress response and signal transduction. In strain MX‐11, several dehydrogenases and cytochrome complexes involved in respiratory chain, including succinate dehydrogenase, l‐proline dehydrogenase, d‐lactate dehydrogenase, l‐lactate dehydrogenase, cytochrome *bc1c* complex, cytochrome *aa3* oxidase, cytochrome *bd* oxidase and nitrate reductase, were upregulated, which would generate a powerful proton‐motive force (Fig. 2C). Likewise, ATP synthase F0F1 subunits responsible for sufficient ATP synthesis using the electrochemical force of the membrane proton gradient were also found to be upregulated, which was expected to provide energy for cell growth.

In MX‐11, transcription of the *narKGHJI* operon encoding a nitrate/nitrite transporter and a respiratory nitrate reductase was enhanced. In the presence of nitrate, *C. glutamicum* can use it as a terminal electron acceptor for anaerobic growth (Nishimura *et al*., 2007). However, CGXII minimal medium is rich in ammonium but contains no nitrate. Considering extra reducing equivalent will be produced during methanol metabolism compared with glucose metabolism, nitrate reduction may be activated under aerobic conditions. Methylisocitrate lyase encoding gene *prpB2* (*cgl0658*) and bifunction citrate synthase/2‐methylcitrate synthase encoding gene *prpC2* (*cgl0659*) also displayed a 3.36‐fold increase and a 6.28‐fold increase in strain MX‐11, respectively. Besides propionate metabolism, these genes are also involved in the formation of citrate and succinate from oxaloacetate, acetyl‐CoA and propionyl‐CoA in *C*. *glutamicum*, which may promote TCA cycle and benefit cell growth (Claes *et al*., 2002; Su *et al*., 2018; Xu *et al*., 2018). There are many other genes with significantly changed mRNA levels, but their roles in methanol‐dependent growth are difficult to interpret due to lack of direct associations with methanol metabolism (Table S1). In the subsequent studies, the effects of nitrate metabolism, amino acid metabolism and the possible SBPase variant pathway on methanol‐dependent growth of *C. glutamicum* were investigated.

### Nitrate reduction by the methanol‐dependent *C. glutamicum*

Nitrate serves as a terminal electron acceptor for the anaerobic growth of *C. glutamicum*, which is attributed to the presence of a respiratory nitrate reductase encoded by the *narKGHJI* gene cluster. The expression of *narKGHJI* operon can only be activated under anaerobiosis and further induced by nitrate (Nishimura *et al*., 2007; Takeno *et al*., 2007). However, the significantly upregulated mRNA levels of *narKGHJI* operon were observed for strain MX‐11 although it was aerobically cultivated. Methanol metabolism is initiated with the oxidation of methanol to formaldehyde by Mdh with the formation of NADH. Therefore, a higher ratio of NADH/NAD^+^ is expected compared with glucose metabolism. In this case, oxygen as a final electron acceptor may be inadequate for balancing the intracellular redox state. Combined with the upregulated *narKGHJI* operon mRNA levels, we hypothesize that the methanol‐metabolizing cells may be deceived by the redox imbalance and activate the nitrate reduction system even they are cultivated under aerobic condition.

**Fig. 3:**
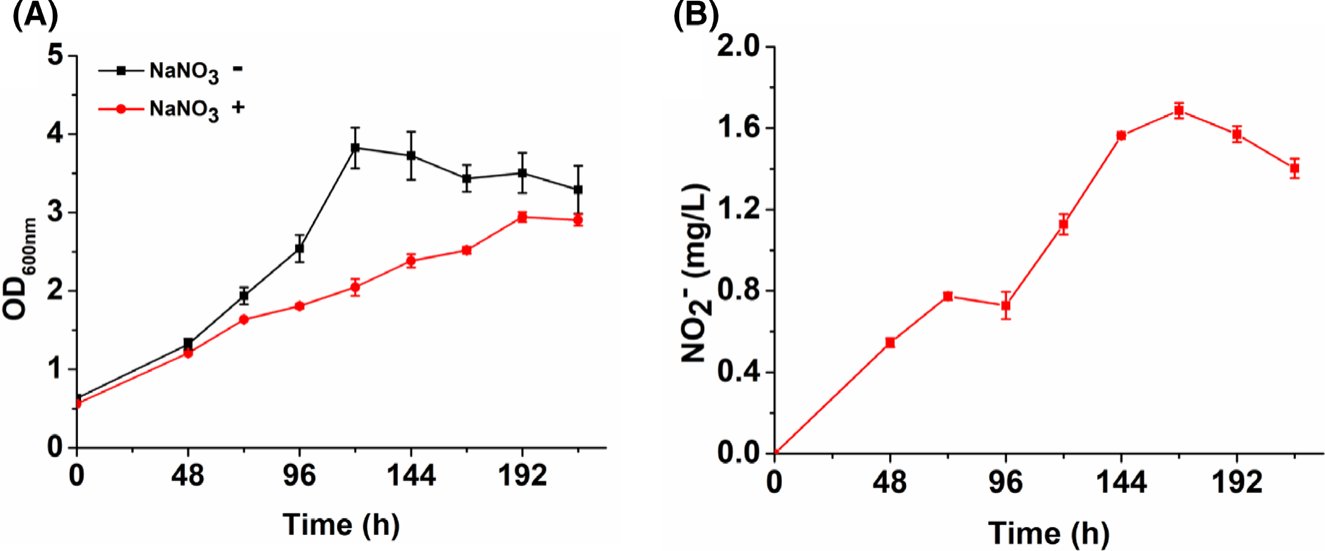
Effect of nitrate addition on growth of strain MX‐11. A. Growth curve of MX‐11 in CGXII medium supplemented with 4 g l^−1^ methanol and 4 g l^−1^ xylose in the presence (red circle) and absence (black square) of 60 mM nitrate. Error bars indicate standard deviations from three parallel experiments (*N* = 3). B. Nitrite formation by MX‐11 supplemented with 60 mM nitrate. Error bars indicate standard deviations from two parallel experiments (*N* = 2).

In order to verify the effect of nitrate metabolism on methanol‐dependent growth of strain MX‐11 under aerobic condition, nitrate was added to CGXII medium supplemented with methanol and xylose as carbon sources. As shown in Fig. 3A, strain MX‐11 exhibited slower growth in the medium containing nitrate and reached to the highest OD_600 nm_ of 2.94 in 192 h, while it grew to a final OD_600 nm_ of 3.82 in 120 h without nitrate. Meanwhile, nitrite, the product of nitrate reduction, was detected in the nitrate‐containing culture and the concentration of nitrite is increased with cell growth, suggesting the activation of nitrate reduction pathway (Fig. 3B). Since *C. glutamicum* does not possess the nitrite ammonification pathway due to the lack of endogenous nitrite reductase (Bonnefoy and Demoss, 1994; Nishimura *et al*., 2007; Takeno *et al*., 2007), the growth defect caused by nitrate addition was possibly due to the accumulation of toxic nitrite (Fig. 3B and Fig. S2). During cultivation with methanol, the shake flasks were covered with a sealing membrane to avoid methanol evaporation, which may affect the oxygen supply. Therefore, the growth of strain MX‐11 without sealing the shaking flasks was measured. The results suggest that strain MX‐11 shows similar cell growth with or without sealing the shaking flasks (Fig. S3). In another study on methylotrophic *E. coli*, nitrate was also added to assist ALE with methanol as a carbon source. The authors suggested that nitrate could serve as an extra electron acceptor but did not detect the nitrite formation (Chen *et al*., 2020). Viewed from another perspective, the activation of the *narKGHJI* operon and reduction of nitrate to nitrite underscore the importance of NAD(H) homeostasis to methanol metabolism. Previous studies have also demonstrated that consuming the excess NADH generated by methanol oxidation or blocking the endogenous NADH formation reactions can improve the methanol bioconversion efficiency in both *in vitro* enzymatic catalysis and *in vivo* methanol metabolism (Price *et al*., 2016; Meyer *et al*., 2018).

### The effect of amino acid supplement on methanol‐dependent growth

**Fig. 4:**
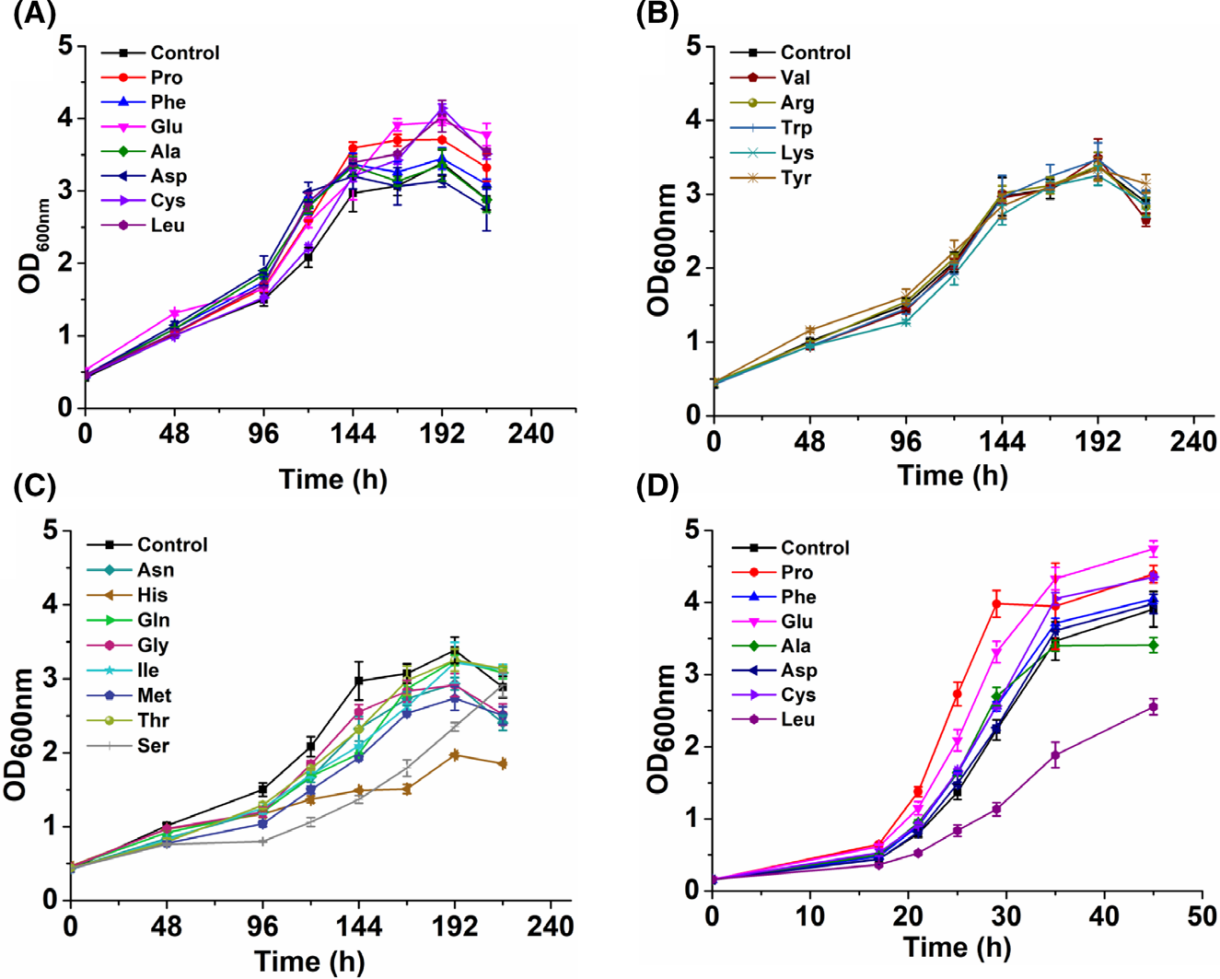
Effect of amino acid supplement on growth of strains MX‐11 and MX‐10. A. Seven amino acids with positive effect on the growth of strain MX‐11 on methanol–xylose medium. B. Five amino acids with no effect on the growth of strain MX‐11 on methanol–xylose medium. C. Eight amino acids with negative effect on the growth of strain MX‐11 on methanol–xylose medium. D. Growth curves of MX‐10 grown on ribose–xylose medium with 5 mM l‐proline, l‐cysteine, l‐glutamate, l‐alanine, l‐aspartate, l‐phenylalanine and l‐leucine, respectively. Error bars indicate standard deviations from three parallel experiments (*N* = 3). Amino acids: l‐alanine (Ala), l‐aspartate (Asp), l‐phenylalanine (Phe), l‐glutamate (Glu), l‐proline (Pro), l‐cysteine (Cys), l‐leucine (Leu), l‐valine (Val), l‐arginine (Arg), l‐tyrosine (Tyr), l‐lysine (Lys), l‐tryptophan (Trp), l‐histidine (His), l‐serine (Ser), l‐methionine (Met), l‐threonine (Thr), l‐isoleucine (Ile), glycine (Gly), l‐glutamine (Gln), l‐asparagine (Asn).

Amino acids are the basic building blocks of proteins, and thus, their biosynthesis and catabolism play crucial roles in cell growth and metabolism. It was observed that the biosynthetic genes of different amino acids were differentially regulated in strain MX‐11, suggesting that the amino acid biosynthesis needs to be reprogrammed to facilitate cell growth on methanol. Therefore, the effect of amino acid supplement on the methylotrophic growth of strain MX‐11 was determined to investigate the potential limitation of biosynthesis of certain amino acids. Twenty proteinogenic amino acids were individually added to CGXII minimal medium supplemented with methanol and xylose for cultivation of strain MX‐11. Significantly increased growth was observed with the addition of l‐alanine, l‐aspartate, l‐phenylalanine, l‐glutamate, l‐proline, l‐cysteine and l‐leucine (Fig. 4A). Supplement of l‐valine, l‐arginine, l‐tyrosine, l‐lysine and l‐tryptophan had no obvious effect on the growth of strain MX‐11 (Fig. 4B). Interestingly, the addition of eight amino acids, such as l‐histidine, l‐serine and l‐methionine, had different levels of negative effect on the growth of strain MX‐11 (Fig. 4C). Amino acid is conventionally considered a growth factor‐stimulating bacterial growth. However, this is not always the case with methylotrophic growth, since negative effect was observed for the addition of eight amino acids.

Regarding the seven amino acids that promote the methanol‐dependent growth of strain MX‐11, it is necessary to investigate whether the growth improvement is associated with methanol metabolism. According to our previous study, the methanol‐dependent strain MX‐10 with *rpiB* deletion also grew using ribose and xylose as co‐substrate (Tuyishime *et al*., 2018). Therefore, we used CGXII minimal medium with ribose and xylose to cultivate the evolved strain MX‐11 and tested the effect of amino acid supplement. However, strain MX‐11 failed to grow with ribose and xylose, which was possibly due to the mutations accumulated during ALE. Genome sequencing of strain MX‐11 revealed one missense mutation (T195I) in UriR, a transcriptional regulator of uridine utilization and ribose uptake genes (Tuyishime *et al*., 2018). The UriR mutation may affect the activation of ribose uptake genes and prevent strain MX‐11 from using ribose as a carbon source (Brinkrolf *et al*., 2008). Instead, we used the ancestral strain MX‐10 to study the effect of amino acid supplement on growth in ribose–xylose medium. l‐Alanine, l‐aspartate, l‐phenylalanine, l‐glutamate, l‐proline, l‐cysteine and l‐leucine that promoted methanol‐dependent growth were individually added to ribose–xylose CGXII minimal medium, and the growth of strain MX‐10 was determined. l‐Glutamate, l‐proline and l‐cysteine enhanced the growth of strain MX‐10, and l‐leucine inhibited cell growth to a great extent (Fig. 4D). Besides, the addition of l‐alanine, l‐aspartate and l‐phenylalanine led to no obvious change in cell growth of strain MX‐10. The results suggest that the growth advantage of strain MX‐11 with the addition of l‐alanine, l‐aspartate, l‐phenylalanine and l‐leucine was directly correlated with methanol metabolism. According to the transcriptome sequencing, the biosynthetic pathway for l‐phenylalanine was upregulated and the downstream utilization pathways of l‐aspartate were weakened, suggesting that cells tended to enhance biosynthesis of these two amino acids (Fig. 2B). Combining the results of amino acid supplement test and the transcriptome analysis, it is suggested that the biosynthesis of l‐alanine, l‐aspartate, l‐phenylalanine and l‐leucine in strain MX‐11 may be a limitation for methylotrophic growth.

Amino acid limitation in methylotrophic *C. glutamicum* is not an exception. It has been reported that the addition of a small amount of yeast extract containing all species of amino acid promoted growth and methanol assimilation of methylotrophic *E. coli* (Whitaker *et al*., 2017). The key contributing amino acid in yeast extract was later identified as l‐threonine (Gonzalez *et al*., 2018). The same group further investigated the effects of activating amino acid biosynthetic pathways on methanol assimilation and methylotrophic growth of *E. coli*. Based on the ^13^C‐methanol labelling data, several amino acids including l‐histidine, l‐isoleucine, l‐leucine, l‐lysine, l‐methionine, l‐phenylalanine, l‐threonine, l‐tyrosine and l‐valine were identified as the limiting amino acids for *E. coli* growth on methanol (Bennett *et al*., 2020a; Bennett *et al*., 2021; Har *et al*., 2021). Although much progress has been made in studying the biosynthesis of limiting amino acids from methanol–carbon in methylotrophic *E. coli*, few studies focus on that in methylotrophic *C. glutamicum*. In this study, biosynthesis of l‐leucine and l‐phenylalanine is also considered as a limitation for methanol‐dependent *C. glutamicum*. However, different from *E. coli*, the biosynthesis of l‐alanine and l‐aspartate from methanol is also limited in *C. glutamicum*. The difference can be explained by the fact that the reported methylotrophic *E*. *coli* was cultivated using methanol and yeast extract containing all species of amino acid, while the methanol‐dependent *C. glutamicum* was cultivated using minimal medium containing methanol and xylose. These findings indicate that the biosynthesis of several amino acids is also a limiting factor for autonomous methylotrophic growth of *C. glutamicum*. Upregulation of the limiting amino acid biosynthesis pathways may be useful to improve methanol assimilation into biomass.

### The cryptic SBPase pathway in *C. glutamicum*

**Fig. 5:**
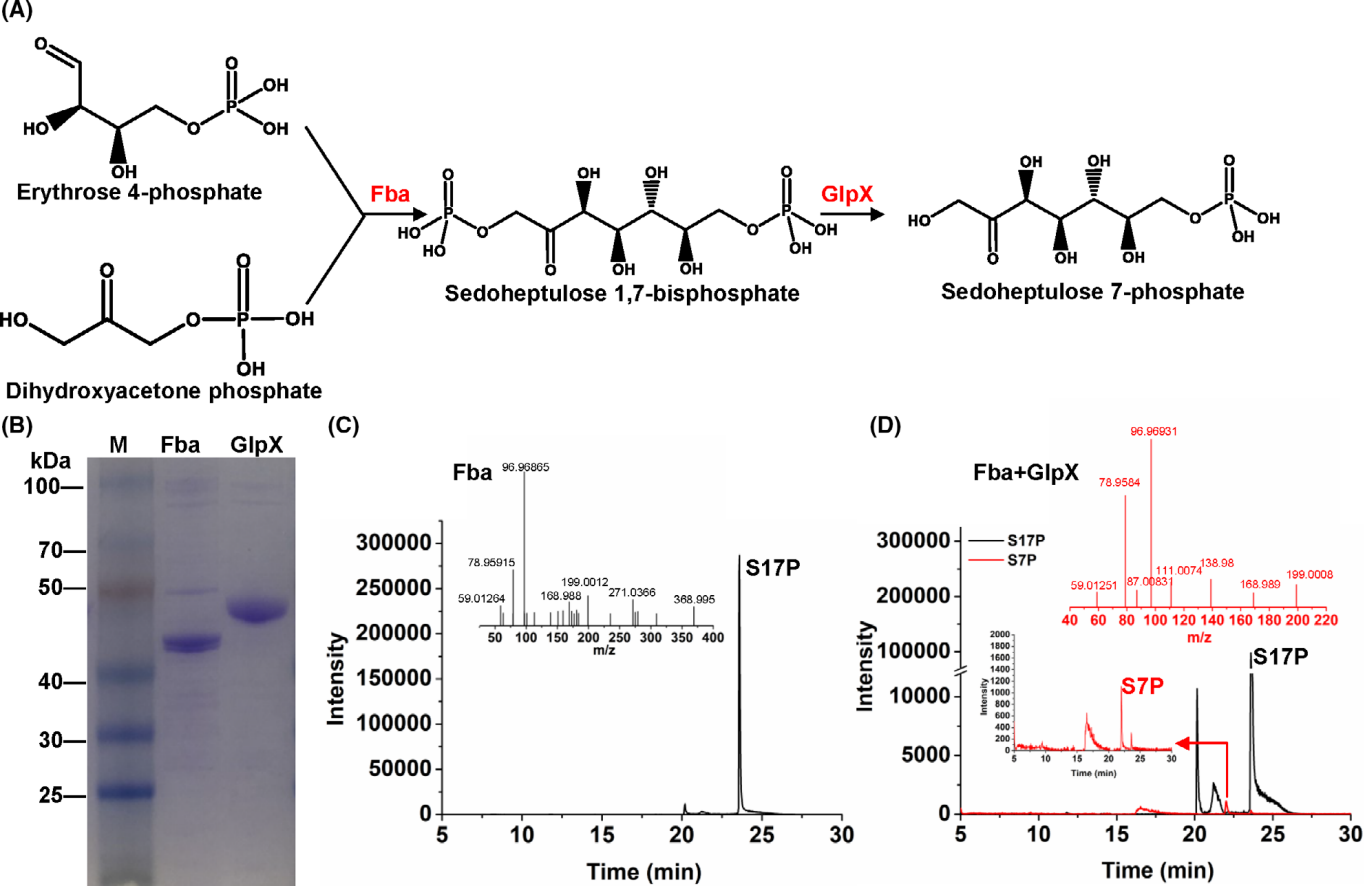
Activity assay of the SBPase variant. A. Cascade reaction for S7P formation from E4P and DHAP catalysed by Fba and GlpX. B. Heterologous expression and purification of GlpX and Fba. C. LC‐MS/MS analysis of S17P formation with purified Fba. D. LC‐MS/MS analysis of S17P and S7P formation with purified Fba and GlpX. Metabolites: Sedoheptulose 1,7‐bisphosphate (S17P), sedoheptulose 7‐phosphate (S7P), erythrose 4‐phosphate (E4P), dihydroxyacetone phosphate (DHAP). Enzymes: fructose bisphosphate aldolase (Fba), bifunction fructose bisphosphatase/sedoheptulose bisphosphatase (GlpX).

Methanol assimilation requires Ru5P as a formaldehyde acceptor, which can be generated by SBPase or Tal variant pathways. In the SBPase variant, E4P and DHAP are condensed to S17P by Fba, and then, the latter is dephosphorylated to S7P by GlpX (Fig. 5A). S7P is then used for Ru5P generation via carbon rearrangement reactions. The dephosphorylation reaction provides driven force for the SBPase variant at an energy cost (Stolzenberger *et al*., 2013). The SBPase variant pathway has not been reported in *C. glutamicum*. Fba and GlpX are considered as glycolytic enzymes in *C. glutamicum* and catalyse the reversible conversion of G3P and DHAP to fructose 1,6‐bisphosphate (F16dP) and the dephosphorylation of F16dP to F6P, respectively. Interestingly, the transcription level of *tal* gene was significantly downregulated, while those of *fba* and *glpX* were upregulated in strain MX‐11. Therefore, it is speculated that a cryptic SBPase variant exists in *C. glutamicum* and is activated during methanol metabolism for efficient generation of S7P and Ru5P.

To test the possibility of SBPase variant in *C. glutamicum*, Fba and GlpX of *C. glutamicum* were heterologously expressed and purified (Fig. 5B). The conversion from DHAP and E4P to S17P and subsequent dephosphorylation to S7P were assayed using enzyme catalysis, and the products were measured using LC‐MS/MS. Without enzyme addition, no product was detected except for the substrates E4P and DHAP. In the presence of E4P, DHAP and purified Fba, S17P was detected in the reaction mixture, but no S7P was detected. When Fba and GlpX were simultaneously added to the reaction mixture, both S17P and S7P were detected (Fig. 5C and D), suggesting the existence of functional SBPase variant in *C. glutamicum*. Considering the downregulation of *tal* and upregulation of *fba* and *glpX*, strain MX‐11 may use the SBPase variant for Ru5P regeneration, which has been proven more favourable for methanol assimilation than the Tal variant. Woolston et al. confirmed that the activation of the SBPase variant in methylotrophic *E. coli* improved methanol assimilation by enhancing regeneration of Ru5P (Woolston *et al*., 2018).

## Conclusions

To characterize the metabolic regulation leading to the improved growth of methanol‐dependent *C. glutamicum*, the evolved mutant MX‐11 and ancestral strain MX‐10 were subjected to transcriptome analysis. Many genes including those of glycolysis, PPP, amino acid biosynthesis and respiratory chain were differentially regulated. Based on the transcriptome analysis, nitrate reduction, amino acid supplement and SBPase variant for Ru5P generation were studied for their roles in methanol‐dependent growth. The results suggest that NADH balance, biosynthesis of several limiting amino acids and Ru5P regeneration are important for improving methanol metabolism by *C. glutamicum*.

## Experimental procedures

### Bacterial strains and growth conditions

The bacterial strains used in this study are listed in Table S2. *E*. *coli* DH5α and *E. coli* BL21 (DE3) were used for general cloning and protein expression, respectively. Cells were cultivated at 37 °C and with shaking at 220 rpm in Luria–Bertani (LB) broth. Ampicillin (100 μg ml^−1^), kanamycin (50 μg ml^−1^) or isopropyl‐β‐d‐thiogalactopyranoside (IPTG, 0.1 mM) was added as required. Methanol‐dependent strains MX‐10 and MX‐11 were cultivated in LBMX medium (LB supplemented with methanol and xylose, 4 g l^−1^ for each carbon source) or CGXIIMX medium (CGXII minimal medium (Keilhauer *et al*., 1993) supplemented with methanol and xylose, 4 g l^−1^ for each carbon source) at 30 °C with shaking at 220 rpm. Cells cultivated in LBMX medium at the mid‐exponential growth phase were harvested, washed twice with CGXIIMX medium and used as a seed culture to inoculate fresh CGXIIMX medium with an initial OD at 600 nm (OD_600 nm_) of 0.5. For transcriptome analysis, bacterial strains were cultivated in CGXIIMX minimal medium at 30°C with shaking at 220 rpm. Cells were harvested at mid‐exponential growth phase and stored at −80 °C before RNA extraction. Kanamycin (15 μg ml^−1^), chloramphenicol (5 μg ml^−1^) and IPTG (1 mM) were added to the medium as required. The shake flasks were covered with a sealing membrane to avoid evaporation of methanol. For amino acid metabolism analysis, 5 mM of a specific amino acid was added except for l‐tyrosine, which was used at 2 mM for its low solubility. CGXIIRX medium (CGXII medium supplemented with ribose and xylose, 4 g l^−1^ for each carbon source) was used for cultivation of MX‐10 with amino acid supplement.

### Measurement of nitrite

Strain MX‐11 was grown on CGXIIMX supplemented with or without 60 mM nitrate. The concentrations of nitrite in cultures were measured using the TNT840‐CN Nitrite Test Kit (HACH, Loveland, CO, USA) with the HACH DR1900 Portable Spectrophotometer (HACH).

### Total RNA isolation and transcriptome analysis

Total RNAs were extracted from cells collected at mid‐exponential phase using RNAprep Pure Cell/Bacteria Kit (Tiangen Biotech, Beijing, China). To minimize DNA contamination, the total RNA samples were treated with RNase‐free DNase I (Tiangen Biotech). RNA quantity and quality were analysed with NanoDrop 2000 Spectrophotometer (Thermo Fisher Scientific, Waltham, MA, USA) by measuring the ratios of A230/260 and A260/280, and RNA integrity was verified by agarose gel electrophoresis. Library construction and transcriptome sequencing on the Illumina HiSeq platform were performed by Novogene Biotech (Tianjin, China). After evaluating the quality of raw sequence reads by FASTQC software (v.0.10.1), NGSQC Toolkit (v.2.3.3) (‐l 70, ‐s 25) was used to filter out low‐quality reads. Next, BWA alignment software (v.0.7.17) was used for aligning the high‐quality reads against the *C. glutamicum* ATCC 13032 reference genome (GenBank Accession Number GCA_000011325.1). The mapping results were sorted and indexed using SAM tools software (v.1.9). Raw read counts from BAM files were obtained using HTSeq (v.0.11.2) software. The raw count table was further processed with the DESeq function of the DeSeq2 package (v.1.18.1) to obtain gene expression data. Genes with a false discovery rate (FDR) < 0.05 and log2(fold change) > 0.5 or < −0.5 were considered as differentially expressed. Pearson’s linear correlation coefficients between variables were calculated using the R package ‘stats’ and plotted using ‘corrplot’. Principal component analysis (PCA) was performed using ‘stats’ package and plotted using ‘ggord’ package.

### Expression and purification of Fba and GlpX

*fba* and *glpX* genes were amplified from the genomic DNA of *C. glutamicum* ATCC 13032 using the primer pairs Fba‐F/Fba‐R and GlpX‐F/GlpX‐R, respectively (Table S3). The *fba* fragment was inserted between the NdeI and XhoI sites of pET‐21a(+) and fused with a C‐terminal His Tag using the ClonExpress II One Step Cloning Kit (Vazyme Biotech, Nanjing, China). The *glpX* fragment was inserted into pET‐28a(+) and fused with a N‐terminal His Tag in the same way. The recombinant plasmid was transformed into *E. coli* BL21(DE3) for protein expression. The resultant strains were cultivated in LB medium containing 100 μg ml^−1^ ampicillin or 50 μg ml^−1^ kanamycin at 37 °C with shaking at 220 rpm. When the OD_600 nm_ reached 0.6–0.8, protein expression was induced by the addition of 0.1 mM IPTG. Cells were then cultivated at 16 °C for another 12 h before they were harvested, washed twice and resuspended with 100 mM potassium phosphate buffer (pH 7.4). Then, cells well disrupted by sonication in an ice bath and the lysed cells were centrifuged at 10 000 *g* for 30 min at 4 °C. The supernatant was used for enzyme purification with a His‐Trap column (GE Healthcare, Pittsburgh, PA, USA) at 4 °C. Purified enzymes were visualized by SDS‐PAGE, and protein concentration was determined with the BCA Protein Assay Kit (Thermo Fisher Scientific, USA).

### Product assay of enzymatic catalysis with Fba and GlpX

The formation of S17P and S7P by Fba and GlpX was determined by a coupled enzymatic assay. The assay mixture contained 100 mM potassium phosphate buffer (pH 7.4), 1 mM DHAP, 1 mM E4P. The reaction was started by the addition of 50 μg purified Fba (S17P formation) or 50 μg purified Fba and 50 μg purified GlpX (S7P formation). A control without enzyme addition was also conducted. The mixture was incubated at 30 °C, and the reaction was stopped at 1 h. S7P and S17P were detected by liquid chromatography coupled with tandem mass spectrometry (LC‐MS/MS) according to the procedure described previously (Wang *et al*., 2020a).

## Conflict of interest

The authors declare no conflict of interest.

## Author contributions

YW, PZ and JS conceived and initiated the project. LF and YW designed the experiments. LF, JQ, NG, ZZ and LS carried out the experiments. LF, YW, XN and QY analysed the data. LF and YW wrote the initial manuscript draft, and all authors contributed to discussion and writing of the final manuscript.

## Supporting information


**Table S1**. Gene transcript level changes between *C. glutamicum* strains MX‐11 vs. MX‐10 cultivated with 4 g l^−1^ methanol and 4 g l^−1^ xylose.

**Fig. S1**. Evaluation of the accuracy and repeatability of transcriptome analysis and classification of differentially expressed genes. (A) Pearson’s correlation coefficient test. (B) Principal component analysis (PCA). (C) Classification of differentially expressed genes according to KEGG_small_class annotation.
**Fig. S2**. Effect of nitrate addition on growth of strain MX‐11. The growth of MX‐11 in CGXII medium supplemented with 4 g l^−1^ methanol and 4 g l^−1^ xylose in the presence (red circle) and absence (black square) of 60 mM nitrate. Error bars indicate standard deviations from three parallel experiments (*N* = 3).
**Fig. S3**. The growth curve of MX‐11 in CGXII supplemented with 4 g l^−1^ methanol and 4 g l^−1^ xylose in shake flasks with (black square) and without (red circle) a sealing membrane. Error bars indicate standard deviations from three parallel experiments (*N* = 3).
**Table S2**. Strains and plasmids used in this study.
**Table S3**. Primers used in this study.
