# Supplementary material for: Transcriptome analysis reveals the roles of nitrogen metabolism and sedoheptulose bisphosphatase pathway in methanol‐dependent growth of Corynebacterium glutamicum
Source: Microb Biotechnol. 2021 Jun 16;14(4):1797–808. doi: 10.1111/1751-7915.13863 (PMC8313271; doi:10.1111/1751-7915.13863)
Supplement: Supplementary file 2 — Fig. S1. Evaluation of the accuracy and repeatability of transcriptome analysis and classification of differentially expressed genes. (A) Pearson’s correlation coefficient test. (B) Principal component analysis (PCA). (C) Classification of differentially expressed genes according to KEGG_small_class annotation. Fig. S2. Effect of nitrate addition on growth of strain MX‐11. The growth of MX‐11 in CGXII medium supplemented with 4 g l−1 methanol and 4 g l−1 xylose in the presence (red circle) and absence (black square) of 60 mM nitrate. Error bars indicate standard deviations from three parallel experiments (N = 3). Fig. S3. The growth curve of MX‐11 in CGXII supplemented with 4 g l−1 methanol and 4 g l−1 xylose in shake flasks with (black square) and without (red circle) a sealing membrane. Error bars indicate standard deviations from three parallel experiments (N = 3). Table S2. Strains and plasmids used in this study. Table S3. Primers used in this study. [file MBT2-14-1797-s002.docx]

**Supplementary information for**

Transcriptome analysis reveals the roles of nitrogen metabolism and sedoheptulose bisphosphatase pathway in methanol-dependent growth of *Corynebacterium glutamicum*

Liwen Fan,^1,2^ Yu Wang,^2,3*^ Jin Qian,^2,4^ Ning Gao,^2,3^ Zhihui Zhang,^2,3^ Xiaomeng Ni,^2^ Letian Sun,^2,3^ Qianqian Yuan,^2^ Ping Zheng^1,2,3**^ and Jibin Sun^2,3^

^1^ *School of Life Sciences, University of Science and Technology of China, Hefei 230026, China.*

^2^ *Key Laboratory of Systems Microbial Biotechnology, Tianjin Institute of Industrial Biotechnology, Chinese Academy of Sciences, Tianjin 300308, China.*

^3^ *University of Chinese Academy of Sciences, Beijing 100049, China.*

^4^ *College of Biotechnology, Tianjin University of Science and Technology, Tianjin 300457, China.*

For correspondence: ^*^E-mail [wang_y@tib.cas.cn](mailto:wang_y@tib.cas.cn); Tel./Fax +86 22 84861943. ^**^E-mail [zheng_p@tib.cas.cn](mailto:zheng_p@tib.cas.cn); Tel./Fax +86 22 84861994.

**
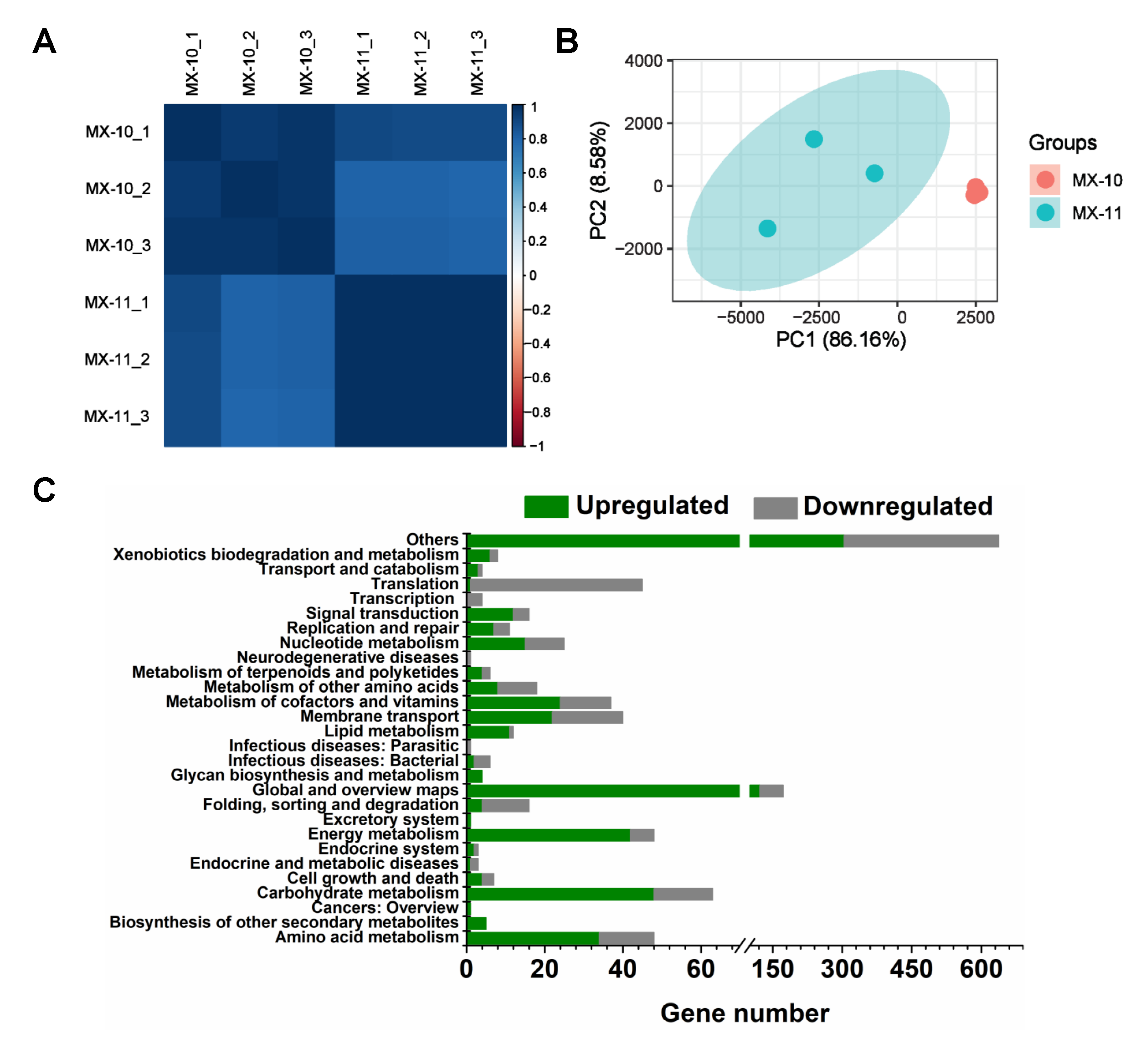
**

**Fig. S1.** Evaluation of the accuracy and repeatability of transcriptome analysis and classification of differentially expressed genes.

A. Pearson’s correlation coefficient test.

B. Principal component analysis (PCA).

C. Classification of differentially expressed genes according to KEGG_small_class annotation.

**

**

**Fig. S2.** Effect of nitrate addition on growth of strain MX-11. The growth of MX-11 in CGXII medium supplemented with 4 g/L methanol and 4 g/L xylose in the presence (red circle) and absence (black square) of 60 mM nitrate. Error bars indicate standard deviations from three parallel experiments (N = 3).

**

**

**Fig. S3.** The growth curve of MX-11 in CGXII supplemented with 4 g/L methanol and 4 g/L xylose in shake flasks with (black square) and without (red circle) a sealing membrane. Error bars indicate standard deviations from three parallel experiments (N = 3).

**Table S1.** Gene transcript level changes between *C. glutamicum* strains MX-11 vs. MX-10 cultivated with 4 g/L methanol and 4 g/L xylose. Table S1 is provided in a separate Excel file.

**Table S2.** Strains and plasmids used in this study

| **Strain or plasmid** | **Description^a^** | **Reference or source** |
| --- | --- | --- |
| **Strain** |  |  |
| *E. coli* |  |  |
| DH5α | General cloning host | TaKaRa |
| BL21 (DE3) | Gene overexpression host | Novagen |
| BL21 (pET-28a-g*lpX*) | Derivative of BL21 (DE3) harboring pET-28a-*glpX* for heterogeneous expression of g*lpX* from *C. glutamicum* fused with a N-terminal His·Tag | This study |
| BL21 (pET-21a*-fba*) | Derivative of BL21 (DE3) harboring pET-21a-*Fba* for heterogeneous expression of *fba* from *C. glutamicum* fused with a C-terminal His·Tag | This study |
| *C. glutamicum* ATCC 13032 | Wild-type strain | ATCC |
| MX-10 | Strain ATCC 13032 harboring pXMJ19-*xylA* and pEC-XK99E-*mdh_Bs_*_2334_-*hps*-*phi_Bm_* with *ald*, *adhE*, *rpiB* gene deleted | (Tuyishime et al., 2018) |
| MX-11 | Mutant of strain MX-10 that grows fast using methanol and xylose as carbon sources | (Tuyishime et al., 2018) |
| **Plasmid** |  |  |
| pET-21a(+) | Overexpression vector, C-terminal His·Tag, Amp^R^ | Novagen |
| pET-28a(+) | Overexpression vector, C-terminal and N-terminal His·Tag, Km^R^ | Novagen |
| pEC-XK99E | Expression vector, IPTG-inducible promoter *P_trc_*, Km^R^ | (Jakoby, et al., 1999) |
| pXMJ19 | Expression vector, IPTG-inducible promoter *P_tac_*, Cm^R^⁠ | (Kirchner and tauch, 2003) |
| pXMJ19-*xylA* | pXMJ19 derivative harboring *xylA* gene from *E. coli*, under the control of *P_tac_* | (Tuyishime et al., 2018) |
| pEC-XK99E-*mdh_Bs_*_2334_-*hps*-*phi_Bm_* | pEC-XK99E derivative harboring *mdh* gene from *Bacillus stearothermophilus* DSM 2334, under the control of *P_trc_*, and *hps* and *phi* genes from *B. methanolicus* MGA3, under the control of constitutive promoter *P_P5_* | (Tuyishime et al., 2018) |

^a^Amp^R^, Km^R^ and Cm^R^ represent resistance to ampicillin, kanamycin, and chloramphenicol, respectively.

**Table S3.** Primers used in this study

| **Primer** | **Sequence (5’-3’)** | **Relevance** |
| --- | --- | --- |
| *fba*-F | gaaggagatatacatatg CCTATCGCAACTCCCGAGGTCTA | pET-21a*-fba* |
| *fba*-R | tggtggtggtggtgctcgag CTTAGAGGTGGTCTTTCCAACAG |  |
| *glpX*-F | gccgcgcggcagccatatg AACCTAAAGAACCCCGAAACG | pET-28a*-glpX* |
| glpX-R | tggtggtggtggtgctcgag TTAGGTCGCGGTGGTGTAGT |  |

**References**

Jakoby, M., Ngouoto-Nkili, C.-E., Burkovski, A. (1999) Construction and application of new *Corynebacterium glutamicum* vectors. *Biotechnol Tech* 13: 437–441.

Kirchner, O., Tauch, A. (2003) Tools for genetic engineering in the amino acid-producing bacterium *Corynebacterium glutamicum*. *J Biotechnol* 104: 287–299.

Tuyishime, P., Wang, Y., Fan, L., Zhang, Q., Li, Q., Zheng, P., *et al*. (2018) Engineering *Corynebacterium glutamicum* for methanol-dependent growth and glutamate production. *Metab Eng* 49: 220–231.
